# Protocol of the EFFORT study: a prospective study of FOLFIRI plus aflibercept as second-line treatment after progression on FOLFOXIRI plus bevacizumab or during maintenance treatment in patients with unresectable/metastatic colorectal cancer

**DOI:** 10.1186/s12885-020-07576-9

**Published:** 2020-11-17

**Authors:** Hironaga Satake, Koji Ando, Eiji Oki, Mototsugu Shimokawa, Akitaka Makiyama, Hiroshi Saeki, Akihito Tsuji, Masaki Mori

**Affiliations:** 1 Cancer Treatment Center, Kansai Medical University Hospital, Hirakata, 573-1191 Japan; 2 Department of Surgery and Science, Graduate School of Medical Science, Kyushu University, Fukuoka, 812-8582 Japan; 3 Department of Biostatistics, Yamaguchi University Graduate School of Medicine, Ube, 755-0046 Japan; 4 Cancer Center, Gifu University Hospital, Gifu, 501-1194 Japan; 5 Department of General Surgical Science Graduate School of Medicine, Gunma University, Maebashi, 371-8511 Japan; 6 Department of Clinical Oncology, Faculty of Medicine, Kagawa University, Miki-cho, 540-0006 Japan

**Keywords:** Colorectal cancer, Aflibercept, FOLFIRI, Second line, FOLFOXIRI, Bevacizumab, Anti-VEGF drug

## Abstract

**Background:**

FOLFOXIRI plus bevacizumab is used as a first-line therapy for patients with unresectable or metastatic colorectal cancer. However, there are no clear recommendations for second-line therapy after FOLFOXIRI plus bevacizumab combination. Here, we describe our planning for the EFFORT study to investigate whether FOLFIRI plus aflibercept has efficacy following FOLFOXIRI plus bevacizumab for mCRC.

**Methods:**

EFFORT is an open-label, multicenter, single arm phase II study to evaluate whether a FOLFIRI plus aflibercept has efficacy following FOLFOXIRI plus bevacizumab for mCRC. Patients with unresectable or metastatic colorectal cancer who received FOLFOXIRI plus bevacizumab as a first-line therapy will receive aflibercept and FOLFIRI (aflibercept 4 mg/kg, irinotecan 150 mg/m^2^ IV over 90 min, with levofolinate 200 mg/m^2^ IV over 2 h, followed by fluorouracil 400 mg/m^2^ bolus and fluorouracil 2400 mg/m^2^ continuous infusion over 46 h) every 2 weeks on day 1 of each cycle. The primary endpoint is progression-free survival (PFS). To achieve 80% power to show a significant response benefit with a one-sided alpha level of 0.10, assuming a threshold progression-free survival of 3 months and an expected value of at least 5.4 months, we estimated that 32 patients are necessary. Secondary endpoints include overall survival, overall response rate, safety, and exploratory biomarker analysis for differentiating anti-VEGF drug in 2nd-line chemotherapy for unresectable or metastatic colorectal cancer.

**Discussion:**

This is the first study to investigate whether FOLFIRI plus aflibercept has efficacy following FOLFOXIRI plus bevacizumab for unresectable or metastatic colorectal cancer. Switching to a different type of anti-VEGF drug in second-line therapy after FOLFOXIRI plus bevacizumab appears to be an attractive treatment strategy when considering survival benefit. It is expected that this phase II study will prove the efficacy of this strategy and that a biomarker for drug selection will be discovered.

**Trial registration:**

Japan Registry of Clinical Trials jRCTs071190003. Registered April 18, 2019.

## Background

One of the goals in chemotherapy for unresectable/metastatic colorectal cancer (mCRC) is to prolong survival and maintain quality of life by controlling the disease through exposure to all active agents in an appropriate sequence of administration. Nine different classes of drugs have shown antitumor activity in mCRC: fluoropyrimidines, irinotecan, oxaliplatin, anti-epidermal growth factor receptor (EGFR) antibodies, anti-vascular endothelial growth factor (VEGF) drugs, regorafenib, trifluridine/tipiracil (FTD/TPI, TAS-102), encorafenib for *BRAF V600E* mutant mCRC, and immunotherapy for microsatellite instability-high/deficient mismatch repair (MSI-H/dMMR) mCRC. Chemotherapy is usually performed with a combination of cytotoxic drugs and a molecular target drug such as anti-VEGF drug or anti-EGFR antibody. A cytotoxic DOUBLET combination of fluorouracil (5-FU) plus levofolinate (l-LV) and either oxaliplatin (FOLFOX) or irinotecan (FOLFIRI) with a molecular target drug is generally proposed as initial systemic chemotherapy; recently, however, a TRIPLET combination of fluorouracil plus levofolinate, oxaliplatin and irinotecan (FOLFOXIRI) showed superior efficacy in terms of tumor shrinkage and survival benefit compared with the DOUBLET combination.

The TRIBE study showed that FOLFOXIRI plus bevacizumab (BEV) is a promising regimen in first–line therapy for patients with mCRC [1], and this regimen is now regarded as a recommended first-line therapy for patients whose treatment goal is tumor shrinkage and in patients with *BRAF* mutant tumors. However, a second-line therapy after FOLFOXIRI plus BEV treatment has not been well established. The TRIBE2 study showed that after maintenance treatment with 5-FU/ l-LV plus BEV, re-introduction of FOLFOXIRI plus BEV offered the most favorable survival benefit [2]. However, most patients who receive an oxaliplatin-based regimen experience peripheral sensory neuropathy. Therefore, FOLFIRI plus BEV appears to be the most commonly used regimen for second-line therapy after FOLFOXIRI plus BEV [1]. Although FOLFIRI plus BEV may be suitable as a standard regimen for second-line therapy, all of the drugs in this regimen are included in first-line FOLFOXIRI plus BEV; accordingly, a response to FOLFIRI plus BEV would not be expected following the failure of first-line FOLFOXIRI plus BEV. Recently, two new anti-VEGF drugs - aflibercept [3] and ramucirumab [4] - showed promising anti-tumor effects as second-line treatment when combined with a FOLFIRI-based regimen for patients with mCRC. FOLFOXIRI plus BEV, or its maintenance phase - 5-FU/ l-LV plus BEV, does not include aflibercept, and thus this drug might provide additional benefit to patients who have progressed after FOLFOXIRI plus BEV.

To investigate this possibility, we planned a phase II EFFORT study to investigate whether FOLFIRI plus aflibercept has efficacy following FOLFOXIRI plus BEV treatment. Here, we describe the protocol for the phase II EFFORT study.

## Methods/design

### Study design and treatment

The EFFORT study is an open-label, multicenter, single arm phase II study to evaluate whether FOLFIRI plus aflibercept has efficacy following FOLFOXIRI plus BEV for mCRC in patients with unresectable or metastatic colorectal cancer. The study has been approved by a central review board and is currently ongoing at 47 medical facilities in Japan. The main inclusion criteria are histologically confirmed advanced mCRC, known *RAS* mutation status (known *BRAF* mutation status also, if possible), mCRC treated with FOLFOXIRI plus BEV as first-line therapy for at least two courses, adjuvant chemotherapy and FOLFOXIRI plus BEV treatment for recurrence, age ≥ 20 years, ECOG PS 0 or 1, measurable lesions based on the Response Evaluation Criteria in Solid Tumors (RECIST) guidelines version 1.1, adequate organ function, and sufficient oral ingestion function. Complete inclusion and exclusion criteria are shown in Table 1. *RAS* and *BRAF* testing are performed locally.

**Table 1:** Patient inclusion and exclusion criteria

Inclusion criteria	
1. Personal written informed consent is obtained after the study has been fully explained	
2. The lead investigator deems that the patient can be treated according to the protocol (the patient is suitable for enrollment)	
3. Histologically confirmed colon or rectal adenocarcinoma	
4. *RAS* mutation analysis at enrollment identifies *RAS* status as either the wild type or mutant type	
5. Patients with unresectable CRC or mCRC who received FOLFOXIRI plus bevacizumab as first-line therapy for at least two courses. First-line therapy is discontinued due to progressive disease (PD) a. Patients with unresectable CRC or mCRC who discontinued first-line therapy with FOLFOXIRI plus bevacizumab	
b. Patients who underwent adjuvant chemotherapy and FOLFOXIRI plus bevacizumab treatment following recurrence (the date of recurrence confirmation should be at least 6 months from the final day of adjuvant therapy.)	
c. Patients who discontinued 5-FU/LV plus bevacizumab as a maintenance therapy (FOLFOXIRI plus bevacizumab as induction therapy will be administered for no more than 12 cycles.)	
7. Age ≥ 20 years at enrollment	
8. ECOG performance status (PS) score of 0 or 1	
9. Measurable lesion in accordance with RECIST ver. 1.1 criteria on contrast-enhanced chest, abdominal, or pelvic (trunk) CT (required within 28 days of enrollment) (Measurable lesions should be 10 mm or more on the major axis using a CT scan with a 5 mm slice. For metastatic lymph nodes, the minor axis should exceed 15 mm in length.)	
10. Patients with sufficient oral ingestion function	
11. Vital organ functions meet the following criteria within 14 days before enrollment. If multiple test results are available in that period, the results closest to enrollment is used. No blood transfusions or hematopoietic factor administration is permitted within 2 weeks before the date on which measurements are taken. a. White blood cell count: ≥3000 /mm^3^, ≤12,000/mm^3^	
b. Neutrophil count: ≥1500/mm^3^	
c. Platelet count: ≥7.5 × 104/mm^3^	
d. Hemoglobin concentration: ≥9.0 g/dL	
e. Total bilirubin: ≤1.5 mg/dL	
f. AST, ALT: ≤100 IU/L (≤200 IU/L for liver metastases)	
g. Serum creatinine: ≤1.5 mg/dL, or creatinine clearance: ≥50 mL/min	
h. Urine protein: ≤1+ (1+ or < 1.0 g/24 h)	
12. Life expectancy ≥3 months	
13. *UGT1A1* polymorphism is wild type or single heterozygous type	
14. Radiation therapy was not administered to the target lesion. However, patients can be included if they: a. Have received neoadjuvant or adjuvant radiation therapy.	
b. Have received radiation therapy against non-target lesions.	
Exclusion criteria	
1. Patients with hypertension (> 160 mmHg systolic or > 100 mmHg diastolic for > 4 weeks) that cannot be adequately controlled with 2 antihypertensive agents* *One antihypertensive treatment containing two antihypertensive agents counts as two antihypertensives.	
2. Patients with diabetes mellitus that cannot be adequately controlled with medication.	
3. Patients with heart disease that may cause problems during the conduct of the study, such as congestive heart failure, angina pectoris requiring medication, clear evidence of transmural myocardial infarction on ECG, clinically evident valvular heart disease, symptomatic coronary disease, poorly controlled arrhythmia, and a previous history of myocardial infarction within the last 12 months.	
4. Patients with severe pulmonary disease, including interstitial pneumonia, pulmonary fibrosis and severe emphysema.	
5. Patients with an active infection.	
6. Patients with clinically significant ascites and pleural effusion.	
7. Patients who have severe drug hypersensitivity (particularly to 5-FU, irinotecan, or aflibercept).	
8. Patients with active multiple cancers. Lesions consistent with carcinoma in situ or intramucosal carcinoma that have been cured by local treatment are not classified as active multiple cancers.	
9. Patients with a psychiatric disorder that may pose a problem, or a history of central nervous system dysfunction.	
10. Patients who have brain metastases.	
11. Patients who have had a gastrointestinal perforation and/or a gastrointestinal fistula up to 6 months prior to enrollment.	
12. Watery diarrhea or diarrhea Grade ≥ 2 at the time of enrollment.	
13. Patients who have had deep vein thrombosis, pulmonary embolism, or some other major form of thromboembolism (portal vein or catheter thrombosis and superficial venous thrombosis qualify as major forms) up to 3 months prior to enrollment.	
14. The patient has experienced any arterial thromboembolic events, including but not limited to myocardial infarction, transient ischemic attack, cerebrovascular accident, or unstable angina, within 6 months prior to the first dose of protocol therapy.	
15. Daily treatment with high-dose aspirin (≥325 mg/day).	
16. Non-steroidal anti-inflammatory medications and immune suppressive or steroidal medications.	
17. Patients receiving phenytoin, warfarin potassium, or flucytosine.	
18. Women who are pregnant, breast feeding, or who wish to conceive.	
19. Men who wish to conceive.	
20. Patients with active gastrointestinal tract bleeding requiring repeated transfusions.	
21. Patients who underwent resection after FOLFOXIRI+bevacizumab because of conversion and experienced disease progression.	
22. Patients who are unable to tolerate aflibercept, 5-FU or irinotecan.	
23. Patients with a severe stenosis due to primary CRC. However, primary patients with CRC resection or colostomy can be included.	
24. Patients with hepatic cirrhosis or active hepatitis.	
25. Patients whom a lead investigator or primary physician deems are not appropriate for this study.	

*One antihypertensive treatment containing two antihypertensive agents counts as two antihypertensives

Patients receive aflibercept and FOLFIRI (aflibercept 4 mg/kg, irinotecan 150 mg/m^2^ IV over 90 min, with l-LV 200 mg/m^2^ IV over 2 h, followed by 5-FU 400 mg/m^2^ bolus and 5-FU 2400 mg/m^2^ continuous infusion over 46 h) every 2 weeks on day 1 of each cycle. Patients receive premedication with antiemetic agents according to institutional guidelines. Treatment continues until disease progression, unacceptable toxicity, death, patient refusal, or investigator decision. When irinotecan is stopped due to severe diarrhea or other adverse events, irinotecan can be skipped, in which case 5-FU/l-LV plus aflibercept or aflibercept alone can be administered. When aflibercept is missed due to an adverse event, FOLFIRI, irinotecan alone, or 5-FU/l-LV can be administered, and such treatments are also within the protocol treatment. There is no prescribed treatment following completion or discontinuation of protocol treatment. Planned enrollment period is 2019 April to 2021 March, and the observation period will include a 2-year follow-up period from the time the last patient is enrolled. No interim analysis of this study will be performed.

### Endpoints and assessments

The primary objective of this trial is to determine whether the FOLFIRI plus aflibercept regimen has efficacy following FOLFOXIRI plus BEV in patients with mCRC in terms of progression-free survival (PFS). Secondary endpoints are overall response rate (ORR), overall survival (OS) and safety.

Disease assessment is performed every 6 weeks by computed tomography (CT). Response is determined by CT scanning based on Response Evaluation Criteria in Solid Tumors version 1.1. PFS is defined as the time from study enrollment to first disease progression or death, whichever occurs first; OS is defined as the time from study enrollment to the date of death due to any cause; and ORR is defined as the percentage of patients relative to the total of enrolled subjects who achieve a complete response (CR) or partial response (PR) based on CT scan images.

All adverse events (AEs) observed during the study treatment period are appropriately registered in the subjects’ medical records and in electronic case reports forms. All serious adverse events (SAEs), namely fatal or life-threatening adverse events or those requiring hospitalization or resulting in persistence or significant disability/incapacity, are required to be disclosed by the investigator to the KSCC (Kyushu Study group of Clinical Cancer) Study Office/Enrollment and Data Analysis Center within 24 h. AEs will be assessed according to the Common Terminology Criteria for Adverse Events version 5.0.

Plasma levels of placental growth factor (PlGF), vascular endothelial growth factor-A (VEGF-A), vascular endothelial growth factor-B (VEGF-B), vascular endothelial growth factor-C (VEGF-C), vascular endothelial growth factor-D (VEGF-D), and interleukin-8 (IL-8) are assessed in blood samples collected from each patient before the protocol treatment, prior to first imaging evaluation, and within 30 days after treatment discontinuation to identify biomarkers that predict the efficacy of aflibercept. This analysis aims to identify a potential predictive biomarker for the efficacy of FOLFIRI plus aflibercept treatment.

### Target sample size and statistical analyses

The primary endpoint of this study is PFS. Second-line PFS with a FOLFIRI-based regimen after FOLFOXIRI/BEV is considered to be shorter than second-line PFS after FOLFOX/BEV due to treatment resistance to irinotecan. According to the TRIBE-2 trial, the second-line PFS of FOLFIRI/BEV after FOLFOX/BEV was 5.6 months [2]. Furthermore, a phase 2 trial of FOLFIRI plus aflibercept conducted in Japan showed its PFS as 5.4 months (95% CI, 4.14–6.70) [5]. Based on these results, the expected PFS value in this study was set at 5.4 months. To achieve 80% power to show a significant response benefit with a one-sided alpha level of 0.10, and assuming a threshold PFS of 3 months, we estimated that 32 patients would be necessary. Considering dropouts, a total of 35 patients would need to be enrolled.

The following hypothesis will be tested using the confidence intervals for median survival time as defined by Brookmeyer and Crowley. Sample size calculation was performed using SAS ver.9.4 (Cary, NC, USA).

## Discussion

A survival benefit for anti-EGFR antibody in 2nd-line chemotherapy has not been shown even in the case of *RAS* wild-type mCRC [6–9]. In contrast, the combination of an anti-VEGF drug and doublet combination has shown a survival effect [3, 4, 10, 11], and an anti-VEGF drug is therefore usually selected in combination with second-line chemotherapy for mCRC. While bevacizumab, aflibercept and ramucirumab are currently used as anti-VEGF drugs [3, 4, 12], no biomarker or definite criteria for selection of these drugs is available, and no data for second-line therapy after FOLFOXIRI plus BEV as pretreatment have been reported.

Attempts to discover a molecular predictive biomarker for anti-VEGF drugs have not led to clinically useful findings, although several studies are currently underway. The acquisition of resistance to BEV in patients with mCRC may involve BEV-induced cytokine changes and high VEGF-A, −D and placental growth factor (PlGF) serum levels [13–16]. Following biomarker analysis of the RAISE trial (NCT01183780), ramucirumab is likely to be added to second-line FOLFIRI for patients with high VEGF-D levels. However, ramucirumab has been reported to show negative effects when administered to patients with low VEGF-D levels equivalent to the one-third of normal levels seen after first-line treatment with a bevacizumab-containing oxaliplatin-based regimen for mCRC [17]. In contrast, a biomarker post hoc analysis of the VELOUR trial (NCT00561470) reported that aflibercept retains its activity regardless of baseline VEGF-A and PlGF levels [18]. The GI-SCREEN CRC-Ukit study, a prospective longitudinal study to investigate an association between plasma angiogenesis-related mediators and clinical outcomes in mCRC, suggested that changes in VEGF-D and PlGF occurred independently, and it may be possible to select drugs by assessing these alterations [19].

Furthermore, regarding the effects of these anti-VEGF drugs, the usefulness of second-line treatment after administration of FOLFOXIRI plus BEV as pretreatment has not been reported. Moreover, the desirability of switching to a different type of anti-VEGF drug in subsequent treatment following BEV as pretreatment remains unclear.

FOLFOXIRI plus BEV showed significant superiority to DOUBLET plus BEV as initial systemic chemotherapy for patients with mCRC in terms of survival benefit and tumor shrinkage [1, 2, 20]. Subgroup analyses also indicated that FOLFOXIRI plus BEV is remarkably effective in patients with poor prognosis, such as those with *BRAF* mutations, extrahepatic metastases or a right-sided primary [1, 20]. Patients with mCRC who receive FOLFOXRI plus BEV as initial systemic chemotherapy may therefore expect an aggressive therapeutic combination as a subsequent regimen. Aflibercept uniquely targets both VEGF-A and PlGF, with higher affinity for both than other anti-angiogenic therapies, and VEGF-A and PlGF bind aflibercept with higher affinity than their native receptor [21]. These findings suggest that tumors progressing under blockade of a single anti-angiogenic therapy, such as BEV, most likely use numerous non-VEGF-A mechanisms to sustain their growth. Switching to a different therapy to target these alternative mechanisms, such as aflibercept, may be beneficial. Although differences in study design and patient characteristics hamper decision-making from cross-trial comparisons, aflibercept plus FOLFIRI combination showed an attractive ORR and survival benefit for patients with mCRC compared to BEV and ramucirumab as second-line therapy (Table 2). This study uses comparison with historical controls to investigate biomarkers able to predict the effects of aflibercept, and to determine the desirability of switching to a different type of anti-VEGF drug for second-line therapy following FOLFOXIRI therapy as pretreatment.

**Table 2:** Background of second-line anti-VEGF drug trials

Trial	ML18147 Ref. [11]		RAISE Ref. [4]		VELOUR Ref. [3]	
Number of cases	820		1072		1226	
Prior oxaliplatin	41.8%		100%		100%	
Prior bevacizumab	100%		100%		30.5%	
Backbone chemotherapy	Irinotecan (35%)		FOLFIRI		FOLFIRI	
Design	Open label		Double blind		Double blind	
Combination	With BEV	Without BEV	With RAM	Without RAM	With AFL	Without AFL
ORR	5%	3%	13.4%	12.5%	19.8%	11.1%
PFS (months)	5.7	4.1	5.7	4.5	6.90	4.67
	HR 0.68	*p* < .0001	HR0.793	*p* < .0005	HR0.758	p < .0001
OS (months)	11.2	9.8	13.3	11.7	13.50	12.06
	HR 0.81	*p* = .0062	HR 0.844	*p* = .0219	HR 0.817	*p* = .0032

*ORR* overall response rate, *PFS* progression-free survival, *OS* overall survival, *BEV* bevacizumab, *RAM* ramucirumab, *AFL* aflibercept

## Data Availability

Not applicable.

## Abbreviations

5-FU: 5-Fluorouracil
AE: Adverse event
BEV: Bevacizumab
CRC: Colorectal cancer
CT: Computed tomography
ECOG: Eastern Cooperative Oncology Group
EGFR: Epidermal growth factor receptor
FOLFIRI: 5-fluorouracil+levofolinate calcium+irinotecan
FOLFOX: 5-fluorouracil+levofolinate calcium+oxaliplatin
FOLFOXIRI: 5-fluorouracil+levofolinate calcium+oxaliplatin+irinotecan
IRI: irinotecan
l-LV: Levofolinate calcium
mCRC: Unresectable/metastatic colorectal cancer
NCCN: National Comprehensive Cancer Network
ORR: Overall response rate
OS: Overall survival
OX: Oxaliplatin
PD: Progressive disease
PFS: Progression-free survival
PS: Performance status
VEGF: Vascular endothelial growth factor
